# Analysis of the Effects of Early Rehabilitation Treatment Conducted by Nurses on the Prevention of Tendon Adhesion after Finger Flexor Tendon Rupture: A Randomized Clinical Trial

**DOI:** 10.1155/2022/8284646

**Published:** 2022-08-09

**Authors:** Sufeng Yin, Xu Sun

**Affiliations:** Department of Hand and Foot Surgery, Taizhou People's Hospital, Taizhou, Jiangsu, China

## Abstract

**Objective:**

This study aims to analyze the application of predictive nursing in the prevention of tendon adhesion, after the anastomosis of finger flexor tendon rupture, so as to provide a basis for clinical nursing.

**Methods:**

A total of 80 patients with anastomoses of flexor tendon ruptures, investigated in our hospital from December 2017 to December 2018, were enrolled in this study. Their data formed the basis of this research. They were divided into two groups, the routine (control) group (*n* = 40) and the nursing group (*n* = 40), by the random number table method. Basic nursing methods only were used in treating the routine group, while the nursing group received basic nursing in combination with early active function exercise. The contrast indices between the two groups were recovery quality of finger flexion and extension, incidence of tendon adhesion, and nursing satisfaction rate.

**Results:**

The probability of tendon rupture and adhesion in the (predictive) nursing group was lower than that found in the control group. The outcomes with predictive nursing were more desirable. The levels of finger flexion and extension in the nursing group were significantly better than those of the control group(*P* < 0.05).

**Conclusion:**

The application of predictive nursing, after the anastomosis of finger flexor tendon rupture, is good for preventing tendon adhesion. Better levels of finger flexion and extension after the operation are guaranteed, and the overall recovery outcomes are better. The satisfaction levels of patients who received predictive nursing were also high, and this method is highly valued and promoted within clinical practice.

## 1. Introduction

The human finger flexor tendon, among its many characteristics, is able to withstand high pressures. However, its metabolism is low, and its elasticity is small. Once a patient has ruptured their finger flexor tendon, anastomosis surgery is necessary, to ensure the most complete recovery of the function possible [1, 2]. However, due to the particular structure of the tissue, adhesion problems can easily occur after the operation, resulting in the procedure failing. Therefore, after operation, it is necessary to implement timely predictive nursing measures for patients. Early active functional exercise, positive mobilization of the enthusiasm of patients in recovery, creates more desirable conditions for postoperative finger function recovery and reduces the risk of reoperation [3–6]. We hypothesized that predictive nursing could prevent tendon adhesion after anastomosis of flexor tendon rupture.

The aim of the study was to highlight the nursing for the prevention of tendon adhesion, by predictive active function exercise in the early stages after the anastomosis of flexor tendon rupture, which will be analyzed, providing a reference for clinical nursing.

## 2. Methods

### 2.1. Study Design

The study was a randomized clinical trial (RCT). The clinical trial registration number is ChiCTR2000035018. This study was conducted with approval from the Ethics Committee of Taizhou People's Hospital (KY201713301). This study was conducted in accordance with the Declaration of Helsinki. Written informed consent was obtained from all participants. A total of 80 patients with anastomoses of flexor tendon ruptures, investigated in our hospital from December 2017 to December 2018, were enrolled in this study. Their data formed the basis of this research. The 80 patients were divided into a routine nursing group (*n* = 40) and a predictive nursing group (*n* = 40). There were 24 men and 16 women in the routine group by the random number table method according to rehabilitation treatment applied. With a median age of (45.00 ± 1.45) years, the youngest was 25 years and the oldest was 65 years. There were 23 men and 17 women in the nursing group. It had a median age of (45.00 ± 1.49) years with a range of 26 years to 64 years. In the two groups, there were 16 cases in zone II, 11 cases in zone III, 6 cases in zone IV, 4 cases in zone V, and 3 cases in other groups. There was no significant difference in the type and site of injury between the two groups. The modified Kessler method was used for suturing. There were no significant differences in gender, age, and Verdan zone between the two groups (*P* > 0.05).

### 2.2. Inclusion and Exclusion Criteria

#### 2.2.1. Inclusion Criteria:

The inclusion criteria were as follows: ① patients with all kinds of hand flexor tendon injuries diagnosed by the clinical medical group; ② patients with complete diagnosis and treatment data; ③ patients with no mental disorders previously or in family history; ④ patients who voluntarily agreed to participate in this research activity and were able to cooperate throughout the process.

#### 2.2.2. Exclusion Criteria

The exclusion criteria were as follows: ① patients with serious and important organ diseases, such as shock and disturbance of consciousness; ② patients with severe tendon injury who need tendon transplantation; ③ patients with were life-threatening symptoms, such as continuous active bleeding.

### 2.3. Study Procedures

#### 2.3.1. Routine Group

The patients are instructed to carry out informed, appropriate activities after their operations. The nursing staff would supervise the patients to carry out appropriate rehabilitation nursing according to the time (routine fasting and drinking before operation, wound nursing, diet guidance, and psychological nursing after operation) to promote wound healing [7, 8].

#### 2.3.2. Nursing Group

Functional exercise plans were made for patients to start soon after their procedures. With the basic activities of the routine group as a foundation, the predictive nursing measures were planned as follows:

1. Preoperation: we need to inform patients, prior to the operation, of the whole procedure and the necessity for early rehabilitation training. This is to help the patients fully understand the causes of tendon adhesion and the damage it can cause at later stages. It will also inform patients of the necessity for engaging in active early rehabilitation exercises, improving the cooperation of the patients, informing the patients of early rehabilitation exercise methods [9, 10], and providing patients with sufficient intervention.
2. Appropriate nursing measures can be provided from 24 hours after operation. The incision of the patient needs to be fully observed within this time frame. The medical dressing is kept dry. Clinicians need to communicate with patients in a timely manner to assess their pain. Patients with severe pain should be treated with analgesia in time. Patients should avoid refusing functional exercise due to pain [11].
3. The week immediately preceding the operation is key for identifying symptoms of adhesion. If pain and swelling are observed within this time, it is necessary to make patients comfortable rather than allowing them to continue the activities unheeded. Nurses used a finger orthosis with a wrist flexion of 20′ 30°, metacarpophalangeal joint flexion of 45°, and interphalangeal joint extension. Passive flexion and active extension exercises were carried out for rehabilitation. 5 flexion movements were completed every hour. Active flexion of interphalangeal joints was prohibited at this stage. At the same time, attention should be paid to the auxiliary flexion and extension of the wrist to avoid stiffness and deformity caused by the flexion of the wrist for a long time [12].
4. Two to three weeks after surgery: the previous actions should be completed, and the range of motion of the flexor tendon of the finger should be gradually increased [13]. At four weeks after the operation, the patient is at an important stage of their recovery. The incision has been healing. Patients wearing finger orthosis could take the initiative to complete mild finger flexion exercises. A set of actions is completed every 2 hours during the day, and each set has completed 5 flexion and extension exercises. The nursing staff could hold the patient's proximal finger, keep the metacarpophalangeal joint in the extension position, and increase the active sliding range of the flexor tendon. After the patients' plaster supports have been removed, they may be instructed to carry out flexion activities. When extending the wrist, the patient should be able to bring the tip of the finger to a distance of 2 cm from the palm. Patients should now be taking the initiative to carry out their own exercises in order to recover their hand function [14, 15].
5. After seven weeks, the patient's plaster slab will have been removed. They should be engaging in active wrist flexion. However, the exercises should be adjusted to compensate for difficulty in bending the wrist. The distance between the finger and palm should now be close to 1 cm. The patient could also use a finger-training device to carry out active exercise and strengthen the antiresistance finger flexion exercise. Basic work such as typing and eating were carried out at 9 weeks after the operation, and fine motor control activities were increased to promote the recovery of finger function. During hospitalization, the rehabilitation nurse was responsible for the follow-up of the whole rehabilitation plan. After discharge, all the patients in the nursing group could effectively implement the supervision measures such as specialist nurse outpatient service, telephone follow-up, and door-to-door extended care [16].

### 2.4. Observational Indices

1. Incidence of tendon adhesion: the numbers and percentages of cases of tendon rupture and tendon adhesion were compared between the groups.
2. Finger rehabilitation efficacy qualified rate: the efficacy was evaluated according to the TAM system standard established by the American Association of Hand Surgery in 1983. The specific method was as follows: the sum of the total active flexion angles of the metacarpophalangeal joint (MP), proximal interphalangeal joint (PIP), and distal interphalangeal joint (DIP) and the sum of the angles with limited extension of these joints were subtracted. The rating is as follows: excellent: the patient's fingers can be normally flexed and extended, and the patient can easily complete eating, typing, and other basic activities (TAM >220°); good: the function of the injured finger recovered to more than 75% of a healthy finger (TAM = 200∼220°); general: the function of the injured finger recovered to 50%∼75% of the healthy finger (TAM = 180∼200°); poor: the function was below 50% of healthy fingers (TAM <180°). The qualified rate of rehabilitation efficacy = (excellent + good + medium) *∗* 100% [17].

### 2.5. Statistical Processing

The data for this study were processed using SPSS20.0 statistical software. The measurement data were expressed as ($\bar{x}$ ± *s*). The Chi-square test was used to compare the ratio between the two groups, and the rank-sum test was used to compare the grade between the two groups. *P* < 0.05 was considered as the significant difference.

## 3. Results

### 3.1. Comparison of Incidences of Tendon Adhesion between the Two Groups

After nursing, the probabilities of tendon rupture and adhesion in the predictive nursing group were lower than in the control group. The outcomes the nursing group provided were more desirable (*P* < 0.05) (see Table 1 for details).

### 3.2. Comparison of Qualified Rate of Curative Effect of Finger Rehabilitation between the Two Groups

The qualified rate of finger rehabilitation efficacy in the nursing group was significantly better than that in the control group, with *P* < 0.05 (see Table 2 for details).

## 4. Discussion

At present, the Chinese rehabilitation physiotherapist industry is in its infancy. Many clinical rehabilitation activities are carried out by rehabilitation nurses. Early use of predictive nursing measures was applied at the early stage to predict potential nursing problems and change passive nursing work into early active intervention to reduce complications. The results of this study showed that the incidence of tendon rupture and tendon adhesion in the nursing group after early adoption of predictive nursing measures is lower than that in the control group, and the nursing effect in the nursing group is more ideal. The finger rehabilitation effect of the nursing group is significantly better than that of the control group (*P* < 0.05). A reason for these findings is that postoperative early recovery is critical for the patients. After a tendon is broken, the synthesis ability of proteins increases. The rate of creation of collagen also increases. Therefore, early rehabilitation training can play a role in the repair of tendon cells and the promotion of tendon healing [18]. In the tendon healing process, the tendon cells display a level of healing ability. Some nutrients in synovial fluid diffusion also promote the healing of incisions. By pursuing early rehabilitation, the blood supply of the tendon area can be restored in time to provide sufficient nutrients for the tendon's recovery. The healing of the incision and nerves is more straightforward. The wound heals along with any damaged nerve, recovering the role of the nervous system in healing, in time, thereby promoting postoperative self-function [19].

This can result in untimely training and exercises being carried out imperfectly. These factors can lead to a significant increase in the number of patients who experience postoperative tendon adhesion. After this operation, the level of the recovery of finger function is not always ideal. Hence, it is necessary to carry out early predictive rehabilitation training in a timely manner, guiding the patients to actively adopt the exercises to promote their continued recovery [20].

Predictive nursing is widely used in clinical practice at present. It is a comprehensive analysis and appraisal of patient needs, using nursing procedures, to allow predictions of potential nursing problems. Passive nursing is transformed into early active nursing interventions that reduce the occurrence of complications, improve nursing quality, and increase patient satisfaction [21]. Nursing staff should understand the postoperative psychological changes of patients who have undergone the repair of the finger flexor tendon. This will help them obtain the trust of patients, enabling them to patiently listen and meticulously see to patient demands. A comprehensive and detailed assessment of the overall state of each patient was carried out. This is a key factor when considering the efficacy of predictive nursing measures. The patients must perform early active exercises, under the guidance of nursing staff, for different time periods. Early active functional exercise, which engages a combination of endogenous and exogenous muscles, should be carried out with the active cooperation of patients. It can reduce tendon adhesion, reduce the contact between anastomosis and surrounding tissues, prevent joint stiffness, and promote the recovery of finger flexor tendons. The study shows that the optimal recovery window for patients with flexor tendon rupture is 2-3 weeks after operation. Timely and effective targeted nursing of patients should be carried out in this key period to achieve maximum recovery with minimal complications [22].

There are several limitations that should be considered. Firstly, this study has a possible selection bias, i.e., only patients aged 25 to 64 years were included in this study. Secondly, the sample size was relatively small, which could decrease statistical power. Anyway, more patients with flexor tendon rupture are recruited to validate our conclusions.

To sum up, early targeted predictive nursing measures are good at preventing tendon adhesion after flexor tendon rupture anastomosis. High levels of finger flexion and extension can be guaranteed after operation, when these measures are taken. The overall recovery outcomes are better. The satisfaction rates of patients receiving predictive nursing are also high. The relationships between nurses and patients have improved, which is an ideal nursing measure. At the same time, the standard of support provided by the clinical nurses is also improved, highly worthy of promotion within the sector.

## Figures and Tables

**Table 1:** Comparison of the incidence of tendon adhesion between the two groups (*n* (%)).

Groups	Tendon rupture	Tendon adhesion	Incidence
Routine group (*n* = 40)	4 (10.00)	10 (25.00)	14 (35.00)

Nursing group (*n* = 40)	2 (5.00)	2 (5.00)	4 (10.00)

Total	6	12	

*X* ^2^	—	—	7.169

*P*	—	—	0.007

*Note.* The figures outside the brackets refer to the number of cases, and the figures inside the brackets refer to the percentage of the total number.

**Table 2:** Qualified comparison of finger rehabilitation efficacy between two groups (*N* (%)).

Groups	Excellent	Good	General	Poor	Qualified rate of finger rehabilitation
Routine group (*n* = 40)	5 (12.50)	15 (37.50)	10 (25.00)	10 (25.00)	30 (75.00)
Nursing group (*n* = 40)	16 (40.00)	6 (15.00)	15 (37.50)	3 (7.50)	37 (92.50)
Total	21	21	25	13	
*Z*	—		—	—	−2.046
*P*	—		—	—	0.041

*Note.* The figures outside the brackets refer to the number of cases only, and the figures inside the brackets refer to the percentage of the total number.

## Data Availability

The datasets used and/or analyzed during the current study are available from the corresponding author on reasonable request.
